# Noninvasive assessment of kidney dysfunction in children by using blood oxygenation level-dependent MRI and intravoxel incoherent motion diffusion-weighted imaging

**DOI:** 10.1186/s13244-021-01091-6

**Published:** 2021-10-21

**Authors:** Ping Liang, Yaxian Chen, ShiChao Li, Chuou Xu, Guanjie Yuan, Daoyu Hu, Ihab Kamel, Yu Zhang, Zhen Li

**Affiliations:** 1 Department of Radiology, Tongji Hospital, Tongji Medical College, Huazhong University of Science and Technology, 1095 Jiefang Avenue, Wuhan, 430030 Hubei China; 2 Department of Pediatrics, Tongji Hospital, Tongji Medical College, Huazhong University of Science and Technology, 1095 Jiefang Avenue, Wuhan, 430030 Hubei China; 3 Russell H. Morgan Department of Radiology and Radiological Science, The Johns Hopkins Medical Institutions, 601 N Caroline St, JHOC 4240, Baltimore, MD 21287 USA

**Keywords:** Blood oxygenation level-dependent magnetic resonance imaging, Intravoxel incoherent motion diffusion-weighted imaging, Chronic kidney disease, Children

## Abstract

**Objectives:**

To explore whether multiparametric approach including blood oxygenation level-dependent MRI (BOLD-MRI) and intravoxel incoherent motion diffusion-weighted imaging (IVIM-DWI) can be applied in the assessment of renal function in children with chronic kidney disease (CKD).

**Materials and methods:**

This prospective study included 74 children (CKD stage 1–3, 51; CKD stage 4–5, 12; healthy volunteers, 11) for renal MRI examinations including coronal T2WI, axial T1WI and T2WI, BOLD-MRI, and DWI sequences. We measured the renal cortex and medulla T2*, ADC, *D*_*t*_, *D*_*p*_, and *f*_*p*_ values on BOLD and DWI images. Appropriate statistical methods were applied for comparing MRI-derived parameters among the three groups and calculating the correlation coefficients between MRI-derived parameters and clinical data. Receiver operating characteristic (ROC) curves were used to assess the diagnostic performance of MRI-derived parameters.

**Results:**

There were significant differences in cortex T2*, ADC, *D*_*t*_, *f*_*p*_ and medulla T2*, ADC, *D*_*t*_ among the three groups. Cortex T2*, ADC, *D*_*t*_, *f*_*p*_ and medulla T2*, ADC, *D*_*t*_ had a trend: CKD stage 4–5 < CKD stage 1–3 < healthy volunteers. Cortex and medulla T2*, ADC, *D*_*t*_ were significantly correlated with eGFR, serum creatinine (Scr), cystatin C. In addition, cortex T2* and eGFR showed the highest correlation coefficient (*r* = 0.824, *p* < 0.001). Cortex *D*_*t*_ and medulla T2* were optimal parameters for differentiating healthy volunteers and CKD stage 1–3 or CKD stage 4–5 and CKD stage 1–3, respectively.

**Conclusions:**

BOLD-MRI and IVIM-DWI might be used as a feasible method for noninvasive assessment of renal function in children with CKD.

## Key points

- Cortex T2*, ADC, Dt, fp and medulla T2*, ADC, Dt had a trend: CKD stage 4–5 < CKD stage 1–3 < healthy volunteers.
- Cortex and medulla T2*, ADC, Dt were all significantly correlated with eGFR, serum creatinine, cystatin C.
- Cortex Dt and medulla T2* were optimal parameters for differentiating healthy volunteers and CKD stage 1–3 or CKD stage 4–5 and CKD stage 1–3, respectively.
- BOLD-MRI and IVIM-DWI might be used as a feasible method for noninvasive assessment of renal function in children with CKD.

## Introduction

Chronic kidney disease (CKD) is recognized as a common disease globally. The worldwide prevalence of CKD is estimated to be 13.4%, and patients with end-stage renal disease (ESRD) requiring renal replacement therapy are estimated between 4.902 and 7.083 million [1]. The global incidence of CKD in children continued to increase and the incidence rate of renal replacement therapy among 0–19 years rose 5.9% between 2000 and 2008 [2]. Therefore, it is meaningful and important to focus on the CKD research in children. The capability to identify children with progressive CKD will be of great significance in clinical practice and prognosis. The estimated glomerular filtration rate (eGFR) is the most commonly used clinical indicator to evaluate the renal function of bilateral kidney. However, the eGFR represents the gross renal function and is not sensitive to the early stages of CKD [3].

There are many causes for the progression of CKD, among which chronic hypoxia in the kidney tissue plays a vital role in CKD and may be the ultimate common pathway leading to ESRD [4]. Therefore, early detection of kidney oxygenation and appropriate management (such as controlling fluid load and avoiding hypotension to improve tissue hypoxia) may contribute to preventing the progression of CKD. Currently, the most common way to monitor kidney hypoxia is to insert microelectrodes directly into the kidney to assess the partial pressure of oxygen [5]. However, this method is invasive and not feasible for follow up due to the inadequate sampling.

Blood oxygenation level-dependent magnetic resonance imaging (BOLD-MRI), which does not need for the introduction of contrast agents or ionizing radiation, can noninvasively assess renal oxygenation level due to the paramagnetic property of deoxyhemoglobin, which resulting in a reduction in the signal on T2*-weighted image. A higher T2* value indicates more oxygen content in the kidney tissue. Therefore, T2* may be able to indirectly characterize the partial pressure of oxygen in the local tissues of the kidney [6, 7].

Persistent hypoxia, damage to the microvascular system and inflammation make the kidneys particularly sensitive to the hypoperfusion and hypoxia damage, resulting in interstitial fibrosis and forming a vicious circle, and finally accelerating the progression of CKD [8]. Percutaneous renal biopsy is still the gold standard method for assessing renal interstitial fibrosis. However, invasive renal tissue biopsy may lead to complications including bleeding, pain, perinephric hematoma, and even acute renal failure [9]. Previous study indicated that the presence of interstitial fibrosis can be detected by applying diffusion-weighted imaging (DWI) [10]. DWI is a non-invasive, non-contrast, and non-radiation imaging method and can reflect the movement of water molecules by the ADC value. However, not only the movement of pure water molecules will affect the ADC value, but also blood perfusion and renal tubule flow will interfere with the ADC value. Intravoxel incoherent motion diffusion-weighted imaging (IVIM-DWI), which was demonstrated a feasible technology for noninvasive evaluation of kidney function and the pathological status of CKD [11], can evaluate both the tissue diffusion and capillary perfusion.

Previous researches about BOLD-MRI and IVIM-DWI are rarely used in children CKD research [12–15]. Therefore, the aim of this research was to investigate whether the multiparametric approach including BOLD MRI and IVIM-DWI can be applied in the assessment of renal function in children with CKD.

## Materials and methods

### Patients

**Fig. 1:**
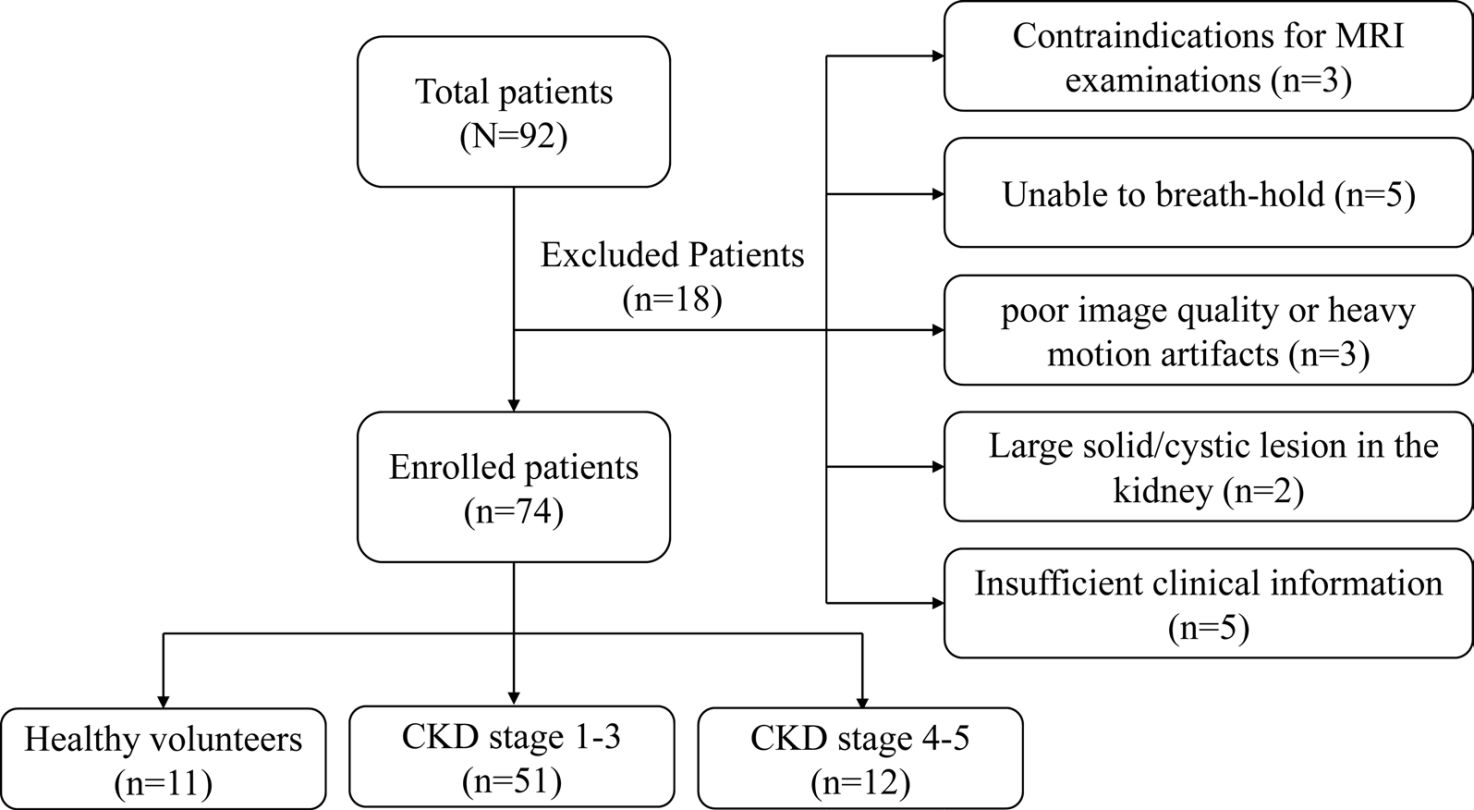
Flowchart of the study population

The Ethics Committee of our hospital agreed the prospective research and all participants including healthy volunteers have signed an informed consent. We prospectively enrolled 81 children with CKD and 11 healthy children from December 2019 to December 2020. The diagnosis of pediatric CKD is based on fulfilling one of the following criteria: 1) GFR of less than 60 mL/min per 1.73 m^2^ for greater than three months with implications for health regardless of whether other CKD markers are present. 2) GFR greater than 60 mL/min per 1.73 m^2^ that is accompanied by evidence of structural damage or other markers of kidney function abnormalities, including proteinuria, albuminuria, renal tubular disorders, or pathologic abnormalities detected by histology or inferred by imaging based on the commandments of Kidney Disease Improving Global Outcomes (KDIGO) of 2012 [16]. Eleven healthy individuals were viewed as controls and these children did not have urinary system diseases, high blood pressure, cardiovascular diseases or diabetes, and did not take any drugs that affect kidney function. The study exclusion criteria were as follows: (1) contraindications for MRI examinations; (2) unable to breath-hold; (3) poor image quality or heavy motion artifacts; (4) Large solid/cystic lesion in the kidney; (5) Insufficient clinical information. The details for excluded patients were shown in Fig. 1. Finally, sixty-three patients (33 men and 30 women, age ranging from 4 to 18 years, mean years 9.60 ± 3.17 years) and eleven healthy children (8 men and 3 women, age ranging from 4 to 13 years, mean years 7.46 ± 2.58 years) were included in this research.

### Study protocol

The MRI examination were performed within one week before the kidney biopsy. All patients and healthy volunteers were instructed to fast for 8 h and water for 4 h before the MRI examination. All children followed our instructions for breathing training, practice holding their breath for a few seconds, or holding their breath during the examination. The MRI examinations were performed on a 3.0 T scanner (Magnetom Skyra, Siemens Healthcare, Erlangen, Germany) with an eighteen-channel phased-array coil. Conventional coronal T2WI, axial T1WI, T2WI and BOLD, DWI sequences were performed. BOLD-MRI examination used multiple gradient echo sequence and was performed on the axial plane. The data acquisition was at the end-expiration using a breath-hold and the parameters were as follows: FOV = 300 × 225 mm, slice thickness = 4.0 mm, Matrix = 192 × 154, TR = 293 ms, No. Echo = 7 equally spaced (2.46–17.22 ms), averages = 1. The acquisition time ranged from 45 to 50 s. DWI applied a single-shot echoplanar imaging (EPI) sequence with free-breathing and combined with reduced volume excitation (ZOOMit) in the axial plane. The parameters were as follows: FOV = 288 × 125 mm, slice thickness = 4.0 mm, Matrix = 120 × 120, TR = 7700 ms, TE = 72 ms. Fat saturation technology can be used to reduce chemical shift artifacts. We applied a 4-directional diffusion-weighting gradient and b-values ranged from 0 to 1000 (0, 20, 50, 80, 100, 200, 500, 800, 1000). A parallel imaging factor was 2 and the acquisition time was about 5–6 min, varying based on the number of slices. The detailed parameters were shown in Table 1.

**Table 1:** MRI acquisition parameters at 3.0 T

Sequence	TR/TE (ms)	FA (°)	Matrix	FOV (mm × mm)	Averages	Slice thickness (mm)	Bandwidth (Hz/Px)	B-values (s/mm^2^)	Acquisition time
T2WI	4950/118	121	384 × 384	280 × 192	2	4.0	303	NA	2 min 30 s
DWI	7700/72	NA	120 × 120	288 × 125	1, 1, 1, 1, 1, 1, 1, 2, 2	4.0	1602	0, 20, 50, 80, 100, 200, 500, 800, 1000	5 min 39 s
BOLD	293/2.46, 4.92,7.38, 9.84,12.30,14.76, 17.22	60	192 × 154	300 × 225	1	4.0	470	NA	54 s

*DWI* diffusion-weighted imaging, *TR/TE* relaxation time/ echo time, *FA* flip angle, *FOV* field of view, *NA* not application

### Image analysis and data measurement

Two radiologists (Xu C and Li Z, with 8 and 18 years of clinical practice in renal MRI, respectively) separately analyzed the images. Neither radiologist knew the patient's clinical information and kidney disease status.

**Fig. 2:**
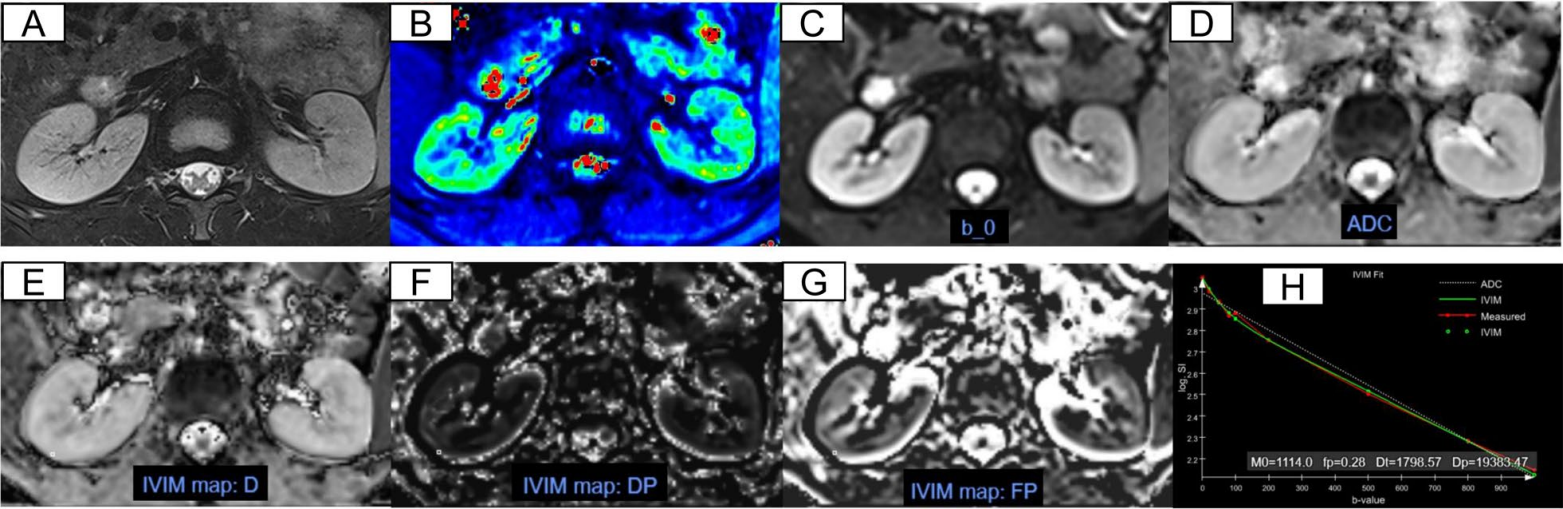
Respective MRI images of healthy volunteers (12 years old, male). **A**–**H** axial T2-weighted images, T2* mapping, b0 map, ADC, *D*_*t*_, *D*_*p*_, *f*_*p*_, IVIM fit, respectively

**Fig. 3:**
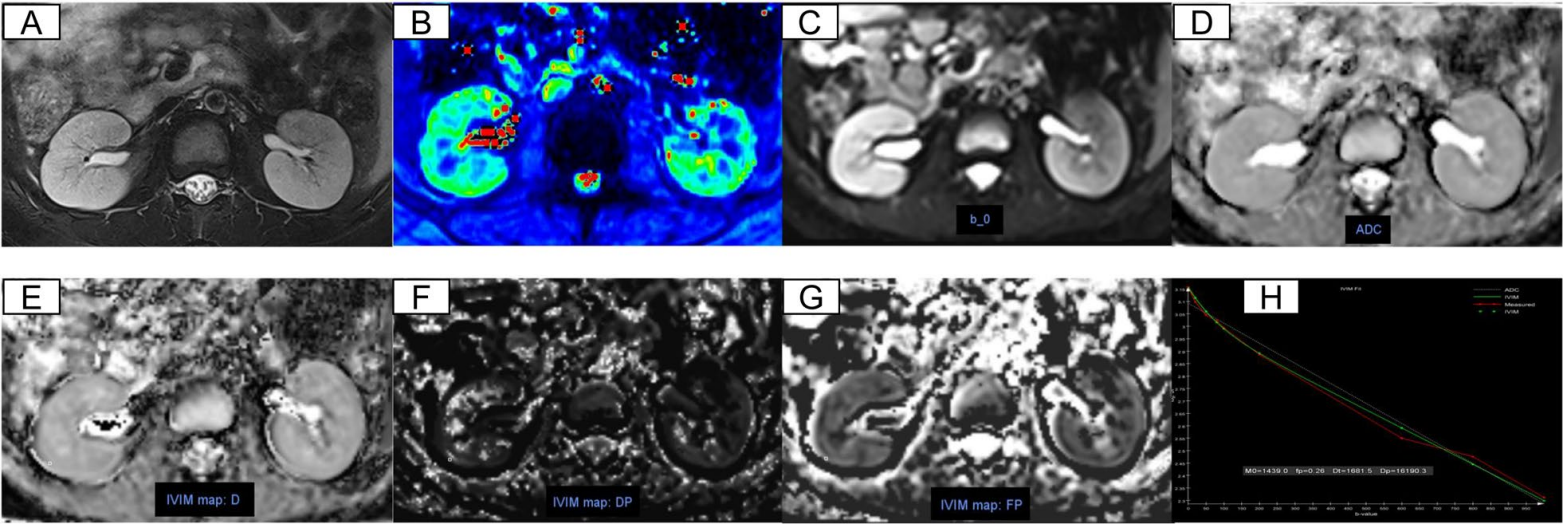
Respective MRI images of CKD stage 1–3 (7 years old, male). **A**–**H** Axial T2-weighted images, T2* mapping, b0 map, ADC, *D*_*t*_, *D*_*p*_, *f*_*p*_, IVIM fit, respectively

**Fig. 4:**
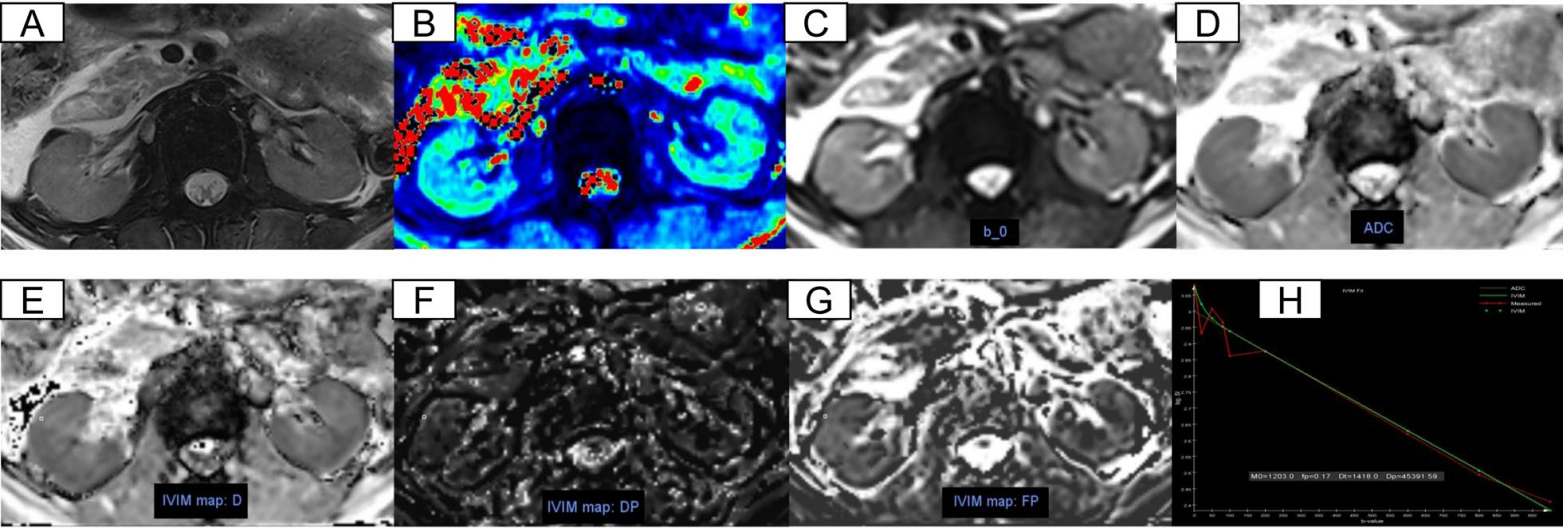
Respective MRI images of CKD stage 4–5 (13 years old, female). **A**–**H** Axial T2-weighted images, T2* mapping, b0 map, ADC, *D*_*t*_, *D*_*p*_, *f*_*p*_, IVIM fit, respectively

BOLD-MRI. After the data acquisitions of this sequence were completed, the images were transferred to the VE40B workstation, and the data was analyzed through the post-processing software. Two radiologists selected an axial slice in the middle of the kidney and manually drew the cortex and medulla regions of interest (ROIs) of the bilateral kidney**s** on the T2*- weighted images to obtain the cortex and medulla mean value of T2*. Cortical ROIs (5–10 cm^3^) were delineated by following the outline of the kidney and 3–4 medulla ROIs (5–10 cm^3^) were selected on the representative slice. The echo time data and signal intensity were fitted to a single decaying exponential function to acquire the value of T2*, which was used as a semi-quantitative index of tissue relative oxygenation. A decrease in T2* indicates an increase in deoxyhemoglobin concentration [17]. Figures 2, 3, 4B shows the representative T2*-weighted images of a healthy volunteer, patients with CKD stage 2 and CKD stage 4.

IVIM-DWI. We used the post processing software offline provided by MR Body Diffusion Toolbox v1.4.0 (Siemens Healthcare) to obtain IVIM-DWI parameters. Freehand cortex and medulla ROIs were delineated bilaterally on renal hilar slice to calculate the mean values of IVIM-DWI parameters. Cortical ROIs (5–10 cm^3^) were delineated by following the outline of the kidney avoiding big lesions, fat and cyst. Medulla ROIs (3–4 ROIs, 5–10 cm^3^) were selected by viewing T2WI anatomical images as a reference standard (Figs. 2, 3, 4A). Three parameters, *D*_*t*_, *D*_*p*_, and *f*_*p*_ were calculated based on a biexponential model: *S*_*b*_/*S*_0_ = (1 − *f*_*p*_) × exp (− *b* × *D*_*t*_) + *f* × exp [–*b* × (*D*_*t*_ + *D*_*p*_)], S0 represents the signal intensity when *b* = 0 s/mm^2^, *S*_*b*_ represents the signal intensity at a specific *b* value, *D*_*t*_ indicates the true diffusion coefficient, *D*_*p*_ indicates the pseudo-diffusion coefficient, and *f*_*p*_ indicates the perfusion fraction. Specifically, ADC values were calculated based on a monoexponential fit using two specific *b* values (0, 1000 s/mm2) according to the equation: *S*_*b*_/*S*_*0*_ = e^−bADCmon^. Figures 2, 3, 4C–H shows the representative DWI images of a healthy volunteer, patients with CKD stage 2 CKD stage 4.

### Clinical data and histopathological evaluation

For all patients, venous blood samples were collected within 10 days before the MRI examination. We measured serum creatinine (Scr) levels, albumin/creatinine ratio (ACR), serum albumin and cystatin C and calculated the eGFR by using the modification of diet in renal disease (MDRD) equation. eGFR = 186 × Scr^−1.154^ × Age^−0.203^ × 0.742 (if female) × 1.233 (if Chinese) [18]. CKD stages were divided into 5 stages according to the value of eGFR recommended by the K/DOQI guideline [19]. CKD1: eGFR > 90 mL/min; CKD2: eGFR = 60–90 mL/min; CKD3: eGFR = 30–60 mL/min; CKD4: eGFR = 15–30 mL/min; CKD5: eGFR < 15 mL/min.

All patients underwent ultrasound-guided kidney biopsies and performed by two renal pathologists within 1–2 days after completing the MR examination in our research. We selected the lower pole of the right kidney as the biopsy position and fewer than 8 glomeruli were not included to the study which was similar to the method used by previous study [20]. All renal biopsy specimens were sent to outside pathology laboratories and read by two pathologists with more than 8 and 10 years of clinical experience in kidney disease. Table 2 recorded the types and numbers of the underlying disease.

**Table 2:** Baseline characteristics

Characteristics	Healthy volunteers	CKD stage 1–3	CKD stage 4–5	*p* value
Sex				0.449
Male	8	27	6	
Female	3	24	6	
Age (years)	7.46 ± 2.58	9.84 ± 2.99	8.58 ± 3.80	0.051
eGFR (mL/min/1.73m^2^)	143.64 ± 24.02	105.67 ± 40.24	15.09 ± 9.16	< 0.001
Serum creatinine (μmol/L)	34.58 ± 8.54	60.40 ± 34.66	462.09 ± 134.59	< 0.001
ACR	13.75 ± 12.64	1644.82 ± 4244.70	5664.08 ± 10,473.95	< 0.001
Serum albumin	45.48 ± 3.52	37.50 ± 9.09	36.84 ± 9.81	0.014
Cystatin C	0.83 ± 0.134	1.25 ± 0.64	4.73 ± 1.75	< 0.001
Underlying disease				
Henoch–Schonlein purpura nephritis	N/A	15	0	N/A
Lupus nephritis	N/A	3	1	N/A
IgA nephropathy	–	12	0	N/A
Nephrotic syndrome	N/A	17	5	N/A
Vesicoureteral reflux	N/A	1	1	N/A
Unknow	N/A	3	5	N/A

Data are expressed as a number (gender and underlying disease) or means ± standard deviations

*eGFR* estimated glomerular filtration rate, *ACR* albumin/creatinine ratio, *N/A* not applicable

### Statistical analysis

SPSS (version 22, Chicago, IL) was used for statistical analysis and the values of *p* < 0.05 were considered significantly different in statistics. We used the Shapiro–Wilk test (*p* ≥ 0.05 demonstrates normal distribution) to assess the normality. Quantitative data was shown as means ± standard deviations. We employed ANOVA to compare the differences of various clinical values and parameters among the three groups. The least-significant difference (LSD) test was applied to find differences between the further two relevant groups. Pearson product-moment correlation coefficient was performed to study the relationship between clinical data and imaging parameters. We calculated the intraclass correlation coefficients (ICCs) to assess the interobserver agreements (0.81–1.00, excellent agreement; 0.61–0.80, moderate agreement; 0.21–0.40, fair agreement; 0.00–0.20, poor agreement).

## Results

### Clinical characteristics

Sixty-three patients with CKD and 11 healthy volunteers were included in the statistical analysis (healthy volunteers, 11; CKD stage 1–3, 51; CKD stage 4–5, 12). There were no significant differences in sex and age among the three groups (*p* = 0.449 and *p* = 0.051, respectively). The values of eGFR, Scr, ACR and cystatin C showed significant differences among the three groups (all *p* < 0.001). The Scr, ACR and cystatin C increased with the decrease of eGFR. Serum albumin showed no significant difference between CKD stage 1–3 and CKD stage 4–5 (*p* = 0.641). Underlying disease types included henoch–schonlein purpura nephritis, lupus nephritis, IgA nephropathy, chronic glomerulonephritis, primary nephrotic syndrome, vesicoureteral reflux, unknow. Table 2 recorded the clinical features of the individuals.

The T2, T2*, ADC, *D*_*t*_, *D*_*p*_, *f*_*p*_ maps of healthy volunteers, CKD stage 1–3 and CKD stage 4–5 were shown in Figs. 2, 3 and 4. Healthy volunteers and CKD stage 1–3 had clear kidney outlines, and the boundary between the renal cortex and medulla was clear. Patients with CKD stage 4–5 had reduced renal cortex thickness and the boundary between cortex and medulla was not clear.

### Interobserver agreement

We applied appropriate statistical methods to calculate the interobserver agreements and the results of all parameters demonstrated excellent repeatability (ICCs ranged from 0.932 to 0.975). Therefore, a measurement result was randomly selected from two radiologists for the statistical analysis. The ICC values for different MRI-derived parameters were present in Table 3.

**Table 3:** The interobserver agreement for different MRI parameters between two radiologists

Parameters	ICC	95% CI
T2*_Cortex_	0.943	0.910–0.964
T2*_Medulla_	0.953	0.926–0.970
ADC _Cortex_	0.932	0.893–0.957
ADC _Medulla_	0.947	0.915–0.966
Dt_Cortex_	0.955	0.928–0.971
Dt_Medulla_	0.940	0.904–0.962
Dp_Cortex_	0.934	0.895–0.958
Dp_Medulla_	0.959	0.934–0.974
fp_Cortex_	0.942	0.908–0.964
fp_Medulla_	0.954	0.926–0.971

ICC, intraclass correlation coefficient; CI, confidence intervals

### Comparisons of renal cortical and medullary MRI-derived parameters

**Fig. 5:**
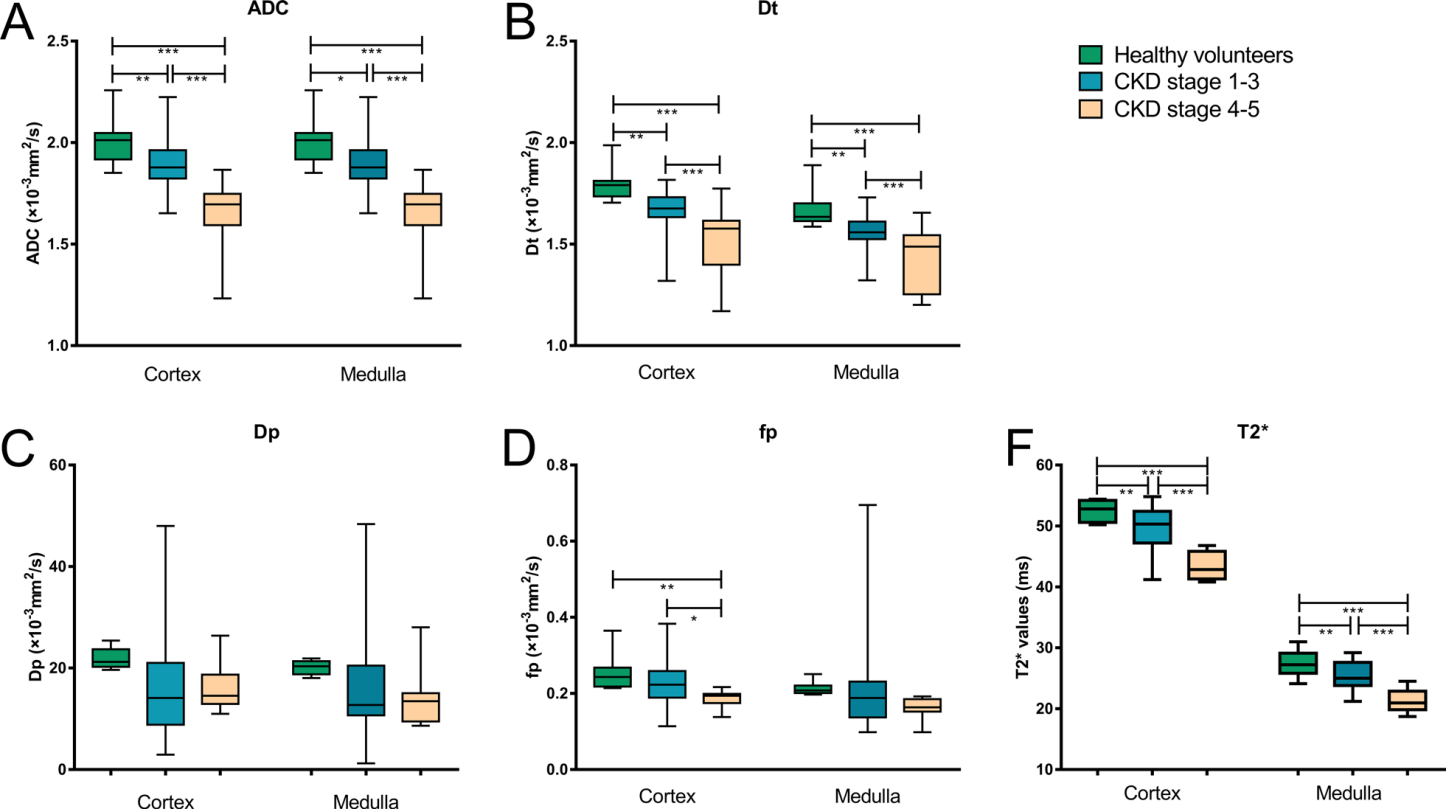
Comparison of MRI-derived parameters between healthy volunteers, CKD stage 1–3 and CKD stage 4–5

There were significant differences in cortex T2*, ADC, *D*_*t*_, *f*_*p*_ and medulla T2*, ADC, *D*_*t*_ among the three groups. Cortex *D*_*p*_ and medulla *D*_*p*_, *f*_*p*_ were not significantly different among the three groups. The values of cortex T2*, ADC, *D*_*t*_, *f*_*p*_ and medulla T2*, ADC, *D*_*t*_ were in the order of healthy volunteers > CKD stage 1–3 > CKD stage 4–5. The results of comparisons were shown in Fig. 5 and Table 4.

**Table 4:** Comparisons of renal cortical or medullary T2*, ADC and IVIM-derived parameters among the three groups

	Healthy volunteers	CKD stage 1–3	CKD stage 4–5	*p* value		
	(*n* = 11)	(*n* = 51)	(*n* = 12)	^a^*P*	^b^*P*	^c^*P*
**Cortex**						
T2*	52.50 ± 1.64	49.67 ± 3.34	43.47 ± 2.31	0.006	< 0.001	< 0.001
ADC	2.014 ± 0.129	1.894 ± 0.118	1.658 ± 0.157	0.006	< 0.001	< 0.001
*D*_*t*_	1.794 ± 0.852	1.670 ± 0.103	1.510 ± 0.178	0.002	< 0.001	< 0.001
*D*_*p*_	21.94 ± 1.882	17.41 ± 10.96	16.35 ± 4.77	0.152	0.159	0.726
*f*_*p*_	0.257 ± 0.051	0.229 ± 0.066	0.187 ± 0.025	0.154	0.006	0.029
**Medulla**						
T2*	27.27 ± 2.16	25.38 ± 2.06	21.33 ± 1.86	0.007	< 0.001	< 0.001
ADC	1.870 ± 0.159	1.752 ± 0.131	1.538 ± 0.146	0.011	< 0.001	< 0.001
*D*_*t*_	1.666 ± 0.086	1.564 ± 0.080	1.432 ± 0.155	0.002	< 0.001	< 0.001
*D*_*p*_	20.06 ± 1.35	15.53 ± 9.83	13.54 ± 5.21	0.239	0.122	0.423
*f*_*p*_	0.214 ± 0.019	0.203 ± 0.096	0.161 ± 0.028	0.694	0.127	0.114

The unit of T2* is ms; ADC, *D*_*t*_ and *D*_*p*_ value are given as × 10^–3^mm^2^/s. The *f* value is dimensionless

*ADC* apparent diffusion coefficient, *D*_*t*_ pure diffusion coefficient, *D*_*p*_ perfusion-related diffusion coefficient, *f*_*p*_ pseudodiffusion fraction

^a^*P* represents Healthy volunteers vs CKD stage 1–3

^b^*P* represents Healthy volunteers vs CKD stage 4–5

^c^*P* represents CKD stage 1–3 vs CKD stage 4–5

### Correlation of clinical data and MRI-derived parameters

**Fig. 6:**
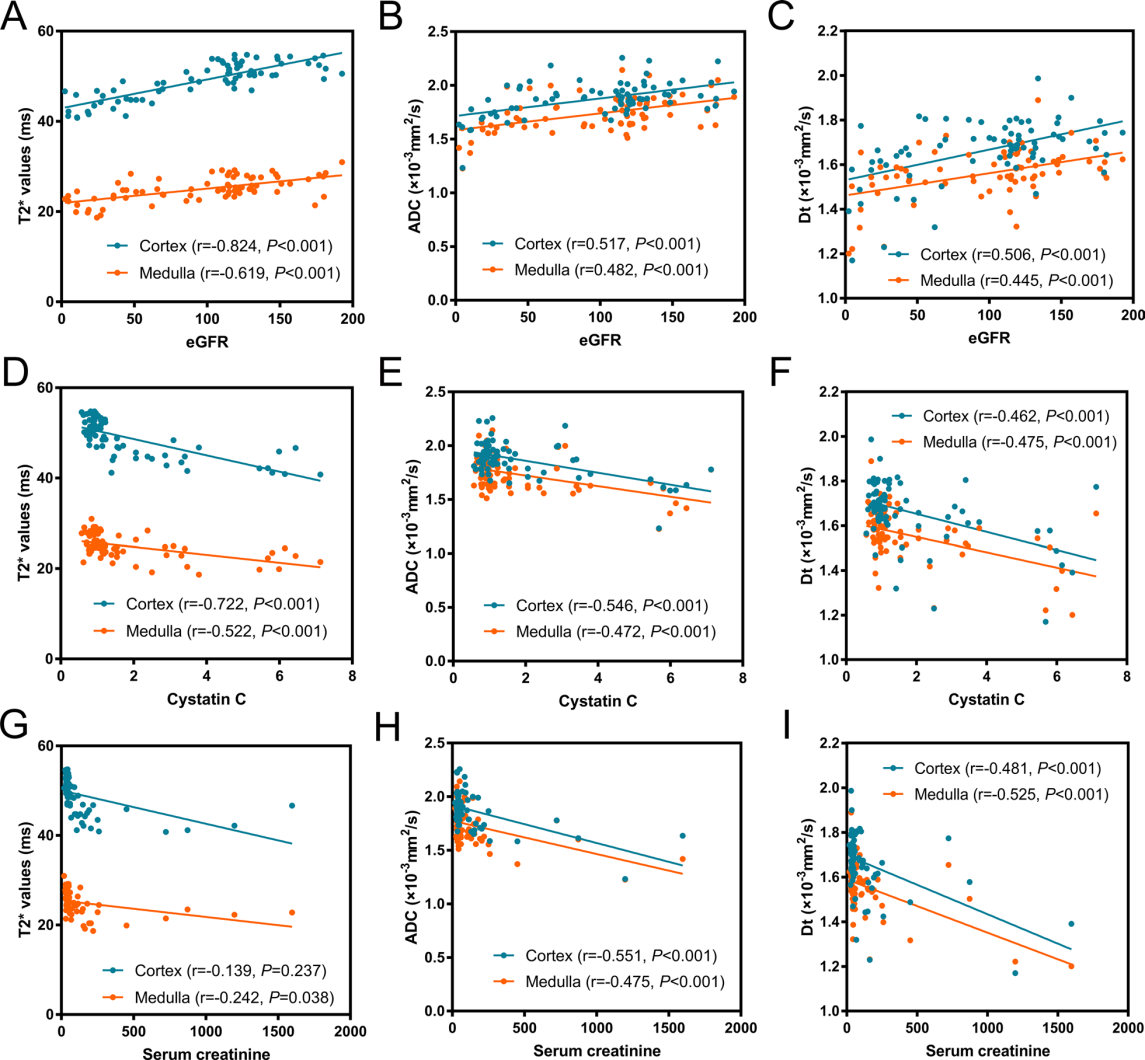
Correlation of clinical data (eGFR, Cystatin C, and serum creatinine) and MRI- derived parameters (T2*, ADC, and *D*_*t*_)

Cortex and medulla T2*, ADC, *D*_*t*_ were all significantly correlated with eGFR, serum creatinine, cystatin C. In addition, cortex T2* and eGFR showed the highest correlation coefficient (*r* = 0.824, *p* < 0.001). The correlations between the clinical data and MRI-derived parameters were presented in Fig. 6 when correlation coefficient was greater than 0.4 (0.8–1.0, very strong correlation; 0.6–0.8, strong correlation; 0.4–0.6, moderate correlation; 0.2–0.4, weak correlation; 0.0–0.2, very weak or no correlation). The correlation of clinical data and MRI-derived parameters were shown in Table 5.

**Table 5:** Correlation of clinical data and MRI- derived parameters

	T2*				ADC				*D*_*t*_	
	Cortex		Medulla		Cortex		Medulla		Cortex	
	*r*	*P*	*r*	*P*	*r*	*P*	*r*	*P*	*r*	*P*
eGFR	0.824	< 0.001	0.619	< 0.001	0.517	< 0.001	0.482	< 0.001	0.506	< 0.001
Serum creatinine	−0.474	< 0.001	−0.350	0.002	−0.551	< 0.001	−0.475	< 0.001	−0.481	< 0.001
ACR	−0.304	0.008	−0.370	0.001	−0.252	0.03	−0.183	0.120	−0.289	0.012
Serum albumin	0.139	0.237	0.242	0.038	0.302	0.009	0.315	0.006	0.289	0.012
Cystatin C	−0.722	< 0.001	−0.522	< 0.001	−0.546	< 0.001	−0.472	< 0.001	−0.462	< 0.001

	*D*_*t*_		*D*_*p*_				*f*_*p*_			
	Medulla		Cortex		Medulla		Cortex		Medulla	
	*r*	*P*	*r*	*P*	*r*	*P*	*r*	*P*	*r*	*P*
eGFR	0.445	< 0.001	−0.091	0.441	0045	0.702	0.007	0.954	0.118	0.316
Serum creatinine	−0.525	< 0.001	0.002	0.984	0.026	0.827	−0.208	0.075	−0.157	0.180
ACR	−0.01	0.934	−0.053	0.654	0.096	0.414	0.150	0.203	−0.077	0.516
Serum albumin	0.104	0.377	0.102	0.385	0.104	0.377	−0.218	0.062	0.115	0.330
Cystatin C	−0.475	< 0.001	0.002	0.988	−0.047	0.690	−0.175	0.136	−0.162	0.168

*eGFR* estimated glomerular filtration rate, *ACR* albumin/creatinine ratio, *ADC* apparent diffusion coefficient, *D*_*t*_ pure diffusion coefficient, *D*_*p*_ perfusion-related diffusion coefficient, *f*_*p*_ pseudodiffusion fraction

The unit of T2* is ms; ADC, *D*_*t*_ and *D*_*p*_ value are given as × 10^–3^mm^2^/s. The *f* value is dimensionless

### Receiver operating characteristic (ROC) curve analysis

**Fig. 7:**
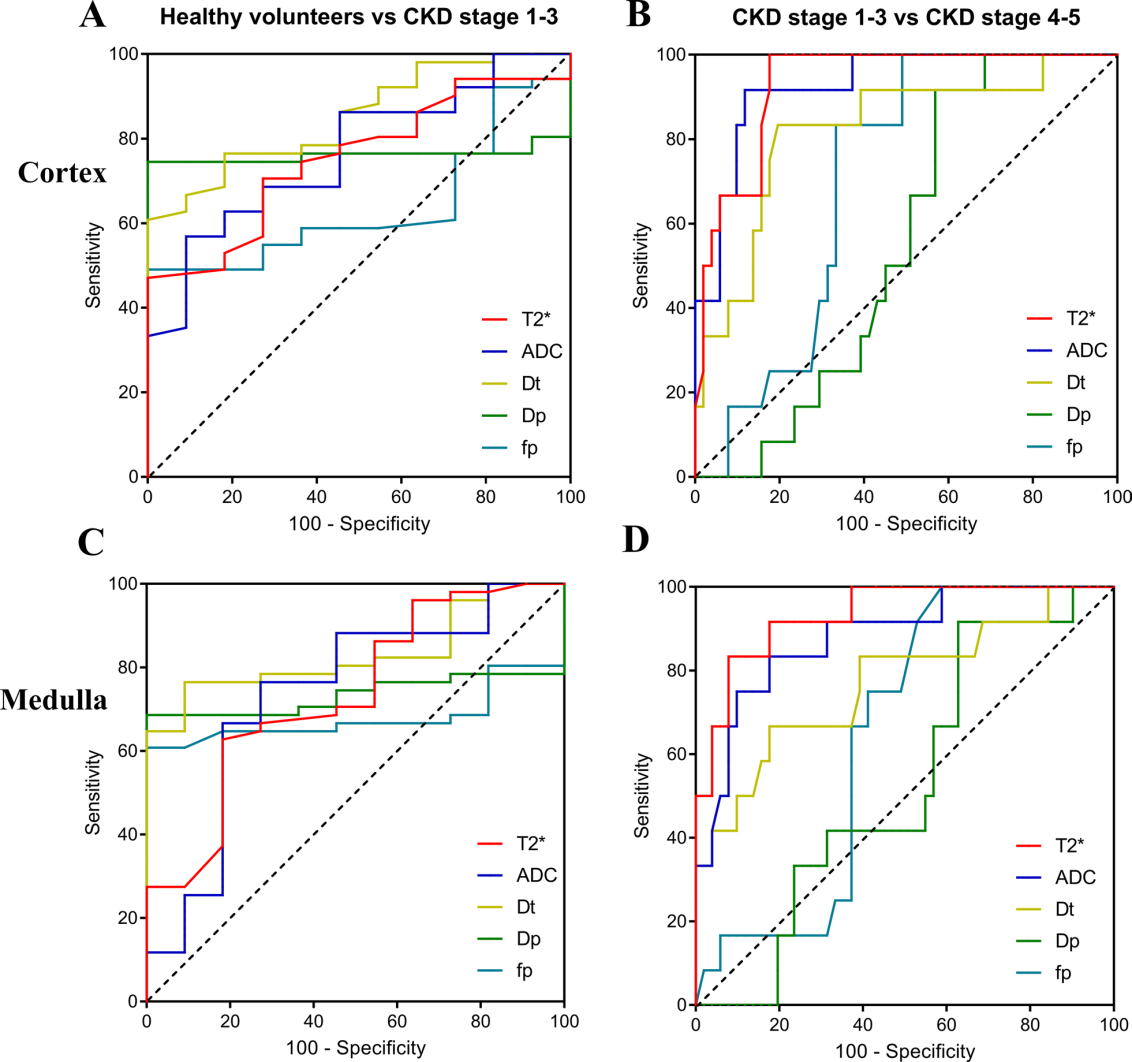
ROC curves for the diagnostic performance of MRI- derived parameters (T2*, ADC, *D*_*t*_, *D*_*p*_, and *f*_*p*_) for differentiating CKD stage 1–3 from helthy volunteers or CKD stage 4–5

To compare the capacity of MRI-derived parameters to differentiate CKD stage 1–3 from healthy volunteers or CKD stage 4–5, ROC curve analysis was performed (Fig. 7, Table 6). Cortex *D*_*t*_ generated the highest area under the curve (AUC, 0.851, 95% CI, 0.738–0.929) for differentiating CKD stage 1–3 from healthy volunteers with sensitivity of 60.78%, specificity of 100%, and a cut-off value of 1.697 × 10^−3^mm^2^/s. Medulla T2* showed the highest AUC (0.935; 95% CI, 0.843–0.981) for differentiating CKD stage 1–3 from CKD stage 4–5 with sensitivity of 92.16%, specificity of 83.33%, and a cut-off value of 22.9 ms.

**Table 6:** The results of ROC analysis including AUC, cut-off, sensitivity, and specific

		Healthy volunteers & CKD stage 1–3				CKD stage 1–3 & CKD stage 4–5			
		AUC (95% CI)	Cut-off	Sensitivity (%)	Specificity (%)	AUC (95% CI)	Cut-off	Sensitivity (%)	Specificity (%)
**Cortex**	T2*	0.752 (0.626–0.853)	49.5	47.06	100	0.931 (0.838–0.980)	46.78	82.35	100
	ADC	0.766 (0.641–0.864)	1.892	56.86	90.91	0.928 (0.834–0.978)	1.78	88.2	91.7
	*D*_*t*_	0.851 (0.738–0.929)	1.697	60.78	100	0.823 (0.706–0.907)	1.616	80.39	83.33
	*D*_*p*_	0.761 (0.636–0.860)	19.33	74.51	100	0.553 (0.422–0.679)	12.75	43.14	91.67
	*f*_*p*_	0.638 (0.506–0.756)	0.213	49.02	100	0.707 (0.578–0.815)	0.217	50.98	100
**Medulla**	T2*	0.735 (0.608–0.839)	25.6	62.75	81.82	0.935 (0.843–0.981)	22.9	92.16	83.33
	ADC	0.736 (0.609–0.840)	1.830	76.47	72.73	0.882 (0.775–0.949)	1.629	82.35	83.33
	*D*_*t*_	0.832 (0.716–0.915)	1.611	76.47	90.91	0.770 (0.647–0.867)	1.509	82.35	66.67
	*D*_*p*_	0.734 (0.607–0.839)	17.96	68.63	100	0.529 (0.399–0.656)	15.29	37.25	91.67
	fp	0.679 (0.548–0.792)	0.193	60.78	100	0.646 (0.516–0.763)	0.192	41.18	100

The unit of T2* is ms; ADC, *D*_*t*_ and *D*_*p*_ value are given as × 10^–3^mm^2^/s. The *f* value is dimensionless

*ROC* receiver operating characteristic, *AUC* area under the receiver operating characteristic curve,* CI* confidence interval, *ADC* apparent diffusion coefficient, *D*_*t*_ pure diffusion coefficient, *D*_*p*_ perfusion-related diffusion coefficient, *f*_*p*_ pseudodiffusion fraction

## Discussion

In this research, we focused on the potential diagnostic performance of T2* relaxation time, ADC, and IVIM MRI-derived parameters in children with CKD. The results of this research showed that T2*, ADC and *D*_*t*_ were significantly related to eGFR, Cystatin C and Scr and enable to assess kidney function in children with CKD. Furthermore, the cortical and medullary parameters obtained from MRI had favorable inter-observer consistency.

BOLD-MRI, which is based on the paramagnetic properties of deoxyhemoglobin and does not need for the introduction of contrast agents, can non-invasively assess the oxygenation of the human kidney tissue [21]. Previous studies showed that T2* derived from BOLD-MRI was positively correlated with eGFR in CKD patients and the T2* values of kidney cortex and medulla in healthy volunteers were significantly larger than those of CKD patients [4]. The results were similar with our study that the cortex or medulla T2* relaxation time was significantly correlated with cystatin C, eGFR and serum creatinine (the absolute value of r was greater than 0.6). Furthermore, the cortical T2* and medulla T2* relaxation time were in the order of healthy volunteers > CKD stage 1–3 > CKD stage 4–5 in our study. Previous studies showed that deoxyhemoglobin can change the spin characteristics of water molecules around the kidney tissue, thereby causing the inhomogeneity of the local magnetic field and shortening the apparent spin relaxation time(T2*) [22, 23]. T2* relaxation time, which was an indicator of the oxygen content of the kidney, was significantly related to the level of deoxyhemoglobin and hypoxia [24]. Our study showed that the value of T2* relaxation time decreased with the development of CKD. The above results showed that T2* was suitable for distinguishing the different stages (healthy volunteers, CKD stage 1–3 and stage 4–5) of CKD patients.

DWI is a promising MRI technology applied to investigate the diffusion and movement of water molecules, reflecting the changes in the microstructure of biological tissues [25]. Previous study showed that the ADC values of healthy volunteers were higher than CKD stage 1–2 and the CKD stage 3 were higher than CKD stage 4–5, however, no obvious difference was found in ADC values between CKD stage 1–2 and CKD stage 3 [26]. This result was similar with our study that the renal cortex and medulla ADC values all had a trend: healthy volunteers > CKD stage 1–3 > CKD stage 4–5, and showed promising value in distinguishing children with CKD. This may be due to the increase in cell density and the presence of fibrosis in the kidney parenchyma, which will lead to an increase in cell membrane density and a decrease in ADC values.

IVIM-DWI, which uses multiple b-values to perform biexponential fitting on DWI, is a more advanced MRI technology compared with the ADC obtained from the monoexponential fitting from DWI and can provide more information about water molecule. IVIM-DWI is able to differentiate between diffusion in extra- and intravascular space: *D*_*t*_ (the true diffusion coefficient), *D*_*p*_ (the pseudo-diffusion coefficient), and *f*_*p*_ (the perfusion fraction). Previous study showed that IVIM-DWI can separate the influence of *F*_*p*_ from true diffusion and improve the evaluation of chronic changes in renal tissue [27].

In our study, cortex and medulla *D*_*t*_ also showed significantly positively correlated with cystatin C, eGFR and serum creatinine (all *r* > 0.4). However, its correlations were not as obvious as ADC values and this result was not consistent with previous research results [28]. However, another research showed that compared with a monoexponential model, the IVIM model had little effect on improving the evaluation of renal insufficiency and this result was consistent with our study [29]. The conflicting results may be caused by the following reasons: first of all, different research groups, Woo S et al. used a rabbit model, adults in Ding et al. study, children in our study; second, the number and size of the b values used were different; Third, different machine models were used. However, they all had the same trend: the values of *D*_*t*_ were in the order of healthy volunteers > CKD stage 1–3 > CKD stage 4–5.

We can further investigate the perfusion-related parameters by IVIM MRI, such *D*_*p*_, *f*_*p*_. These perfusion-related parameters may have important clinical significance because hemodynamic changes occurred during the process of renal interstitial fibrosis formation, such as decreased blood flow of capillaries around renal tubules and changes in vascular endothelial growth factor [30, 31]. In our sresearch, only cortex *f*_*p*_ showed significant differences between healthy volunteers, CKD stage 1–3 and CKD stage 4–5 among these perfusion-related parameters. Previous study showed similar results with our study and demonstrated that *f*_*p*_ had a significant negative correlation with the area of fibrosis, whereas *D*_*p*_ did not show a significant relationship [28]. Bane et al. [32] also showed that IVIM parameters did not correlate significantly eGFR, only cortical and medullary *D*_*t*_ and ADC were positively correlated with contrast-enhanced MRI GFR. In a previous research of CKD patients by Mao et al. [33], both *f*_*p*_ and *D*_*p*_ were significantly negatively correlated with eGFR and the histopathological fibrosis score. Therefore, this indicates that the perfusion-related parameters from IVIM-DWI may be related to the progression of renal fibrosis, but they are currently not robust enough to be used in the clinical setting. The following factors may have contributed to the discrepancies between studies: the different number and size of b-values, and the contrary research groups.

There were several limitations of our study. First of all, this was a single-center research, and the numbers of CKD stage 4–5 were relatively small, which may influence the clinical application of the conclusion. Second, the ROI technique used in this research was the most commonly used clinically but relatively old method. When the kidney function was in good condition, the ROI is easy to place. For patients with ESRD, it was difficult to place ROI due to the lack of visually distinguishable distinction between the renal cortex and medulla. The twelve-layer concentric objects (TLCO) method is currently the most commonly recommended method [23]. Third, kidney BOLD-MRI examination was easily affected by respiration movement, and some younger children cannot hold their breath well during abdominal MRI examinations, although they had carried out breathing training and been put on abdominal belts before the examination.

In conclusion, multiparametric MRI including BOLD MRI and IVIM-MRI, especially T2* relaxation time, ADC and *D*_*t*_ values, might be feasible for noninvasive assessment of renal function in children with CKD.

## Data Availability

The datasets used and/or analyzed during the current study are available from the corresponding author on reasonable request.

## Abbreviations

ACR: Albumin/creatinine ratio
BOLD: Blood oxygenation level-dependent
CKD: Chronic kidney disease
eGFR: Estimated glomerular filtration rate
ESRD: End-stage renal disease
ICC: Intraclass correlation coefficient
IVIM-DWI: Intravoxel incoherent motion diffusion-weighted imaging
ROC: Receiver operating characteristic
ROIs: Regions of interests
Scr: Serum creatinine
